# The efficacy of neuroendoscopic surgery treating patients with thalamic hemorrhage accompanied by intraventricular hematoma

**DOI:** 10.3389/fsurg.2024.1472830

**Published:** 2024-10-28

**Authors:** Feilong Yang, Wuhuan Xu, Xielin Tang, Yan Yang, Buqian A. Ku, Yiping Zhang, Xiaoli Yang, Wei Xie, Xuhui Hui

**Affiliations:** ^1^ Department of Neurosurgery, Santai Hospital Affiliated to North Sichuan Medical College, Mian Yang, Sichuan, China; ^2^ Department of Neurosurgery, West China Hospital, Sichuan University, Chengdu, Sichuan, China; ^3^ Department of Neurology, Santai Hospital Affiliated to North Sichuan Medical College, Mian Yang, Sichuan, China; ^4^ Department of Neurosurgery, Traditional Chinese Medicine Hospital, Le Shan, Sichuan, China

**Keywords:** thalamic hemorrhage, ventricle hemorrhage, surgery, neuroendoscopy, drainage, complication, prognosis thalamic hemorrhage, prognosis

## Abstract

**Objective:**

Neuroendoscopic surgery (NES) has been proven to be safe and effective in hematoma evacuation for cerebral hemorrhage. However, its efficacy for thalamic hemorrhage accompanied by intraventricular hematoma (THAVH) remains unclear. The aim of this study is to determine the efficacy of NES in treating THAVH.

**Method:**

A retrospective study was carried out. The data of patients diagnosed with THAVH were collected from January 1st, 2019, to January 1st, 2022. Patients received the NES or external ventricle drainage (EVD) treatment were assigned to the NES or EVD group, respectively. As primary outcomes, the hematoma evacuation volume, residual hematoma volume, and hematoma clearance rate were separately calculated based on the hematoma site; and the 180-day-mRS score was assessed. As secondary outcomes, the length of stay in the ICU and hospital, and the adverse events were also compared.

**Results:**

Thirty-five patients, aged 66.37 ± 6.62 years, were in the NES group; and 40 patients, aged 68.75 ± 7.22 years, were in the EVD group. The baseline characteristics in the two groups were similar (*P* > 0.05). The gross hematoma evacuation volume, volume of hematoma evacuated in the thalamus or the ventricle, and the hematoma clearance rate were greater in the NES group than in the EVD group on the 1st day after surgery (*P* < 0.05). The patients had a better rank of mRS in the NES group (*P* < 0.05). Compared with patients with mRS > 3, the mean residual hematoma volume in the thalamus of patients with mRS ≤3 on the 1st and 7th day were less in each group (*P* < 0.05), respectively. A residual hematoma volume in the ventricle of patients with mRS ≤3 was less than that of patients with mRS >3 in the EVD group on the 1st day after surgery (*P* < 0.05). GCS score on the 3rd day was greater in the NES group (*P* < 0.05). The incidence of lung infection was lower in the NES group (*P* < 0.05). The length of stay in the ICU and hospitalization duration were shorter in the NES group (*P* < 0.05).

**Conclusions:**

Neuroendoscopic surgery has a greater hematoma clearance rate, a lower lung infection rate and a shorter duration in the hospital. Neuroendoscopic surgery might improve patients’ prognosis. Neuroendoscopic surgery is a safe and effective procedure for treating thalamic hemorrhage accompanied by intraventricular hematoma.

## Introduction

Spontaneous intracerebral hemorrhage (SICH) is a critical subtype of stroke caused by the rupture of small arteries, veins, or capillaries, with a high morbidity, mortality, and disability rates worldwide (1, 2). In China, the mortality of SICH is approximately 40% within 30 days (3). Thalamic hemorrhage is a common but serious deep type of spontaneous cerebral hemorrhage, accounting for 10%–20%, and frequently breaks into the ventricle (4). Concurrent intraventricular hemorrhage is closely associated with high mortality, approximately 60%–70%, in patients with thalamic hemorrhage (5, 6).

Surgery as an option to rapidly remove hematoma has been researched in several trials; minimally invasive surgery has the potential to decrease the mortality of patients with SICH (2). The MISTIE study suggests that a less residual hematoma has a greater potential for good functional outcomes (7). External ventricle drainage (EVD) is routinely adopted for thalamic hemorrhage accompanied by ventricle hemorrhage (THAVH). EVD can decrease intracranial pressure and combining EVD with thrombolytic drugs drains a partial decomposition of blood clots (8). The aggressive clearance of hematoma may improve the poor prognosis of patients (9). However, a drainage catheter should not be left in the ventricle for an extended period due to increasing the risk of intracranial infection. In addition, the catheter is frequently occluded by clots, resulting in insufficient drainage. These factors may result in unsatisfactory efficacy of EVD in improving the prognosis of patients. With the development of minimally invasive techniques, neuroendoscopy is generally used in neurosurgery (10). Neuroendoscope, with good illumination and multi-angle vision, can be used in a long and slender channel (10), which may decrease the extent of iatrogenic injury and potentially improve neurological function outcomes (11). Research shows that NES has a relatively high hematoma evacuation rate and a shorter length of stay in the hospital compared with EVD (10). However, its effectiveness in improving neurological function remains to be validated (12). In addition, a hematoma evacuation in the ventricle or thalamus site was not separately investigated in these researches (4, 10, 13). Hematomas in different sites of the thalamus may result in various outcomes (14). Hence, it is unclear whether the hematoma evacuation rate of the ventricle or thalamus affects patients’ outcomes. Further research is needed to determine the efficacy of NES for patients with thalamic hemorrhage accompanied by intraventricular hemorrhage.

In this retrospective study, we compared the NES with the EVD treatment in terms of hematoma evacuation, residual hematoma, mRS scores and complications. These results may provide pieces of evidence to support the efficacy of NES for patients with THAVH.

## Methods

### Design and participants

A retrospective study was carried out to compare the effects of NES and EVD treating THAVH. Patients diagnosed with spontaneous cerebral hemorrhage were enrolled from January 1st, 2019, to January 1st, 2022. The inclusion criteria were as follows: (1) first diagnosis of spontaneous thalamic hemorrhage accompanied by intraventricular hematoma, and the patients received NES or EVD treatment. (2) Aged 18–80 years. The exclusion criteria were as follows: (1) secondary cerebral hemorrhage, i.e., arteriovenous malformation or aneurysm rupture, or hemostatic disorders associated with intracerebral hemorrhage. (2) Recurrence of cerebral hemorrhage. (3) Patients received routine craniotomy or conservative treatment. (4) Patients whose medical records were incomplete. (5) Patients who had a history of stroke, and (6) patients whose mRS >1 before the onset of the disease. Our study was approved by the ethics committee of Santai Hospital affiliated to North Sichuan Medical College and was carried out following the 1964 Helsinki Declaration and its later amendments.

### Interventions

All patients received therapy based on the Chinese Multidisciplinary Diagnosis and Treatment Guidelines for Spontaneous Cerebral Hemorrhage. The basic treatments were the same in the two groups. We used the Kocher point as the entry point for the NES and the EVD treatment. Under general anesthesia, the patients were placed in the supine position. A linear incision was made on the scalp at the designated entry site. In the NES group, a bony window with a diameter of approximately 3 cm was created; the dura mater was incised in a cross-like pattern. Bipolar cauterization was used to incise the cortex with a diameter of approximately 1 cm. Subsequently, a catheter was inserted toward the anterior horn of the lateral ventricle to establish a surgical corridor. A transparent sheath was inserted along the corridor. Then, an aspirator and a neuroendoscope were introduced into the sheath up to the hematoma cavity under vision on the screen. The hematoma was gradually aspirated through the aspirator by adjusting the position and angle of the sheath and neuroendoscope. Bipolar cautery or hemostatic sponge compression was applied for active bleeding. After clearing the hematoma and stopping the bleeding, a pliable catheter was retained in the hematoma cavity to drain any remaining fluid hematoma. Finally, the bone flap was repositioned and secured, and the scalp was sutured. In the EVD group, a bony hole was created; the dura mater was incised in a cross-like pattern. Bipolar cauterization was used to incise the cortex with a diameter of approximately 2 mm. Subsequently, a catheter was inserted into the anterior horn of the lateral ventricle. Bloody cerebral spinal fluid was gradually drained. Then, the catheter was fixed, and the incision was sutured. After surgery, 50,000 IU of urokinase was injected into the ventricle through the catheter under sterile conditions. The frequency of urokinase use was determined by the residual hematoma.

### Outcomes

The volume of the hematoma was calculated with 3D Slicer software. The hematoma evacuation volume (Ve) was defined as the hematoma volume (Vp) before surgery minus the residual hematoma volume (Vr) at the first brain CT scan after surgery. The hematoma clearance rate (HR) was calculated as (Vp−Vr)/Vp×100%. As primary outcomes, the hematoma evacuation volume, the hematoma clearance rate and the residual hematoma volume were compared on the 1st day after surgery; the prognosis 180 days after disease onset was also assessed. In addition, as secondary outcomes, the adverse events were also compared in the two groups within one week from admission.

### Statistical analysis

All the data were analyzed using the statistical software SPSS (version 26, IMB Corporation). The Kolmogorov‒Smirnov test was applied to determine whether the quantitative data met normality. Quantitative data following normal distribution were described as mean ± standard deviation ($\bar{x}$ ± SD) and compared using an independent sample *t*-test. Quantitative data not following normal distribution were described as the median (P25, P75) and were compared using the Wilcoxon rank sum test. Qualitative data were described as numbers (percentages) and were compared using the Chi-Square test or the Fisher's exact test. The difference was considered to be statistically significant (*P* < 0.05).

## Results

### Patient clinical characteristics

A total of 241 patients were diagnosed with spontaneous thalamic hemorrhage from January 1st, 2019, to January 1st, 2022. Among them, 82 patients diagnosed with thalamic hemorrhage accompanied by intraventricular hematoma received NES or EVD treatment. Seven patients were excluded due to missing follow-up. Hence, 75 patients were recruited in this retrospective study based on the inclusion and exclusion criteria. A total of 35 patients, comprising 19 males and 16 females, with an average age of 66.37 ± 6.62 years, were in the NES group; and 40 patients, comprising 22 males and 18 females, with an average age of 68.75 ± 7.22 years, were in the EVD group. The baseline characteristics between the two groups were similar (Table 1).

**Table 1:** Baseline characteristics of the NES and EVD group.

	Total	NES group	EVD group	*P* value
Age, years	67.64 ± 7.00	66.37 ± 6.62	68.75 ± 7.22	0.143
Sex, *n* (%)				
Male	41 (54.67)	19 (54.29)	22 (55)	0.951
Female	34 (45.33)	16 (44.71)	18 (45)	
Onset time of disease, h	7 (5,8)	7 (5,8)	7 (5,7.75)	0.846
Time of onset to surgery, h	12 (10,14)	13 (10,15)	11 (10,12)	0.061
GCS on admission	8 (8,9)	8 (8,9)	8 (8,9)	0.117
NIHSS on admission	21 (19,22)	22 (20,23)	21 (19,22)	0.289
Volume of hematoma, ml	30.57 ± 2.21	31.00 ± 2.05	30.19 ± 2.30	0.113
Graeb score	9 (8,9)	9 (8,9)	9 (8,9)	0.802
Comorbidities, *n* (%)				
COPD	19 (25.33)	6 (17.14)	11 (27.50)	0.285
CHD	16 (21.33)	8 (22.86)	8 (20.00)	0.763
Diabetes	12 (16.00)	5 (14.29)	7 (17.50)	0.705
Smoking, *n* (%)	16 (21.33)	6 (17.14)	10 (25.00)	0.407
Alcohol consumption, *n* (%)	12 (16.00)	5 (14.29)	7 (17.50)	0.705
RBC, ×10^12^	5.41 ± 0.92	5.30 ± 0.95	5.51 ± 0.89	0.730
WBC, ×10^9^	6.60 ± 2.34	6.70 ± 2.29	6.52 ± 2.40	0.321
PLT, ×10^9^	197.84 ± 19.29	197.2 ± 17.57	198.40 ± 20.88	0.790
APPT	28.97 ± 3.70	29.36 ± 4.43	28.6 ± 2.92	0.339
PT	12.88 ± 1.01	12.78 ± 0.94	12.96 ± 1.06	0.430
INR	1.19 ± 0.21	1.18 ± 0.19	1.20 ± 0.23	0.679
CI	2.55 ± 0.58	2.57 ± 0.71	2.54 ± 0.55	0.787
Serum albumin, g/L	36.39 ± 2.29	36.87 ± 2.59	35.9 ± 1.93	0.092
Blood glucose, mmol/L	6.60 (5.70, 8.50)	6.70 (6.10,7.40)	6.55 (4.65, 9.20)	0.826
Creatinine, mmol/L	63.84 ± 6.08	63.28 ± 6.02	64.3 ± 6.18	0.458

COPD, chronic obstructive pulmonary disease; CHD, coronary heart disease; CI, comprehensive coagulation index; RBC, red blood cell; WBC, white blood cell; PLT, platelet; APPT, activated partial thromboplastin time; PT, prothrombin time; INR, international normalized ratio.

### Hematoma clearance in the two groups

**Figure 1:**
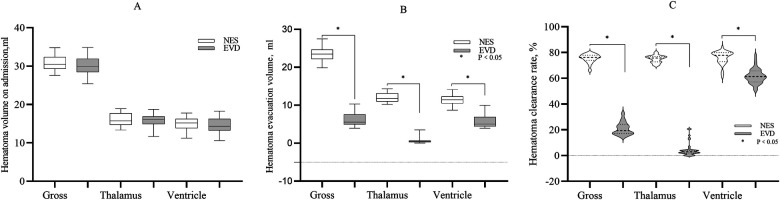
**(A)** The gross hematoma volume and hematoma volume in the thalamus and ventricle on admission. The baseline hematoma volumes were similar in two the groups (*P* > 0.05) **(B)** The gross hematoma clearance volume and the volume of hematoma evacuated in the thalamus or ventricle were greater in the NES group than in the EVD group on the 1st day after surgery (*P* < 0.05) **(C)**. The gross hematoma clearance rate and hematoma clearance rate of the thalamus or ventricle were greater in the NES group than in the EVD group on the 1st day after surgery (*P* < 0.05).

The gross volume of hematoma evacuated in the NES group was greater than that in the EVD group on the day 1st after surgery (23.35 ml vs. 6.33 ml, *P* < 0.05). The volume of hematoma evacuated in the thalamus on the 1st day was greater in the NES group than in the EVD group (11.77 ml vs. 0.42 ml, *P* < 0.05). The volume of hematoma evacuated in the ventricle on the 1st day was greater in the NES group than in the EVD group (11.46 ml vs. 5.05 ml, *P* < 0.05). The hematoma clearance rates were greater in the NES group than in the EVD group on the 1st day after surgery (*P* < 0.05) (Figure 1).

### Prognosis of patients in the two groups

**Figure 2:**
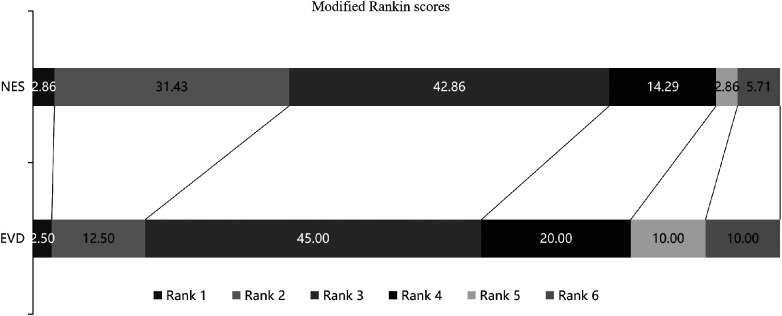
The proportion of patients with scores ranging from 1 to 6 on the mRS. The proportion of patients with mRS ≤3 was77.15% in the NES group, and that was 60% in the EVD group. Patients had a better rank of mRS scores in the NES group than in the EVD group (*P* < 0.05).

Within 6 months of follow-up, there were 1, 11, 15, 5, 1, and 2 patients in the NES group and 1, 5, 18, 8, 4, and 4 patients in the EVD, with scores ranging from 1 to 6 on the mRS. Patients had a better rank of mRS scores in the NES group than in the EVD group (*P* < 0.05). Among them, 2 and 4 patients died in the NES and the EVD groups, respectively. The difference in mortality between the two groups was not statistically significant (*P* > 0.05) (Figure 2; Table 2).

**Table 2:** Complications, death, GCS score and re-operation in the two groups.

	Total	NES group	EVD group	*P* value
Lung infection, *n* (%)	33 (44.00)	11 (31.43)	22 (55.00)	0.040
Urinary tract infection, *n* (%)	14 (18.67)	5 (14.29)	9 (22.50)	0.362
Intracranial infection, *n* (%)	5 (6.67)	3 (8.57)	2 (5.00)	0.637*
DVT, *n* (%)	12 (16.00)	4 (11.43)	8 (20.00)	0.312
IMVT, *n* (%)	22 (29.33)	9 (25.71)	13 (30.50)	0.520
Re-bleeding, *n* (%)	5 (6.67)	1 (2.86)	4 (10.00)	0.364
Hydrocephalus within 6 months, *n* (%)	7 (9.33)	2 (5.71)	5 (12.50)	0.438
Epilepsy, *n* (%)	5 (6.67)	3 (8.57)	2 (5.00)	0.637*
Cerebral infarction in hospitalization, *n* (%)	3 (4.00)	2 (5.71)	1 (2.50)	0.596
Re-operation, *n* (%)	3 (5.33)	1 (2.86)	3 (7.50)	0.618*
GCS on 3rd day	10 (9, 11)	11 (10, 11)	10 (9, 11)	0.033
Death within 6 months, *n* (%)	6 (8.00)	2 (5.71)	4 (10.00)	0.679*

DVT, deep venous thrombosis; IMVT, intramuscular venous thrombosis.

* Fisher's extract test.

### The mRS score and the residual hematoma volume

**Figure 3:**
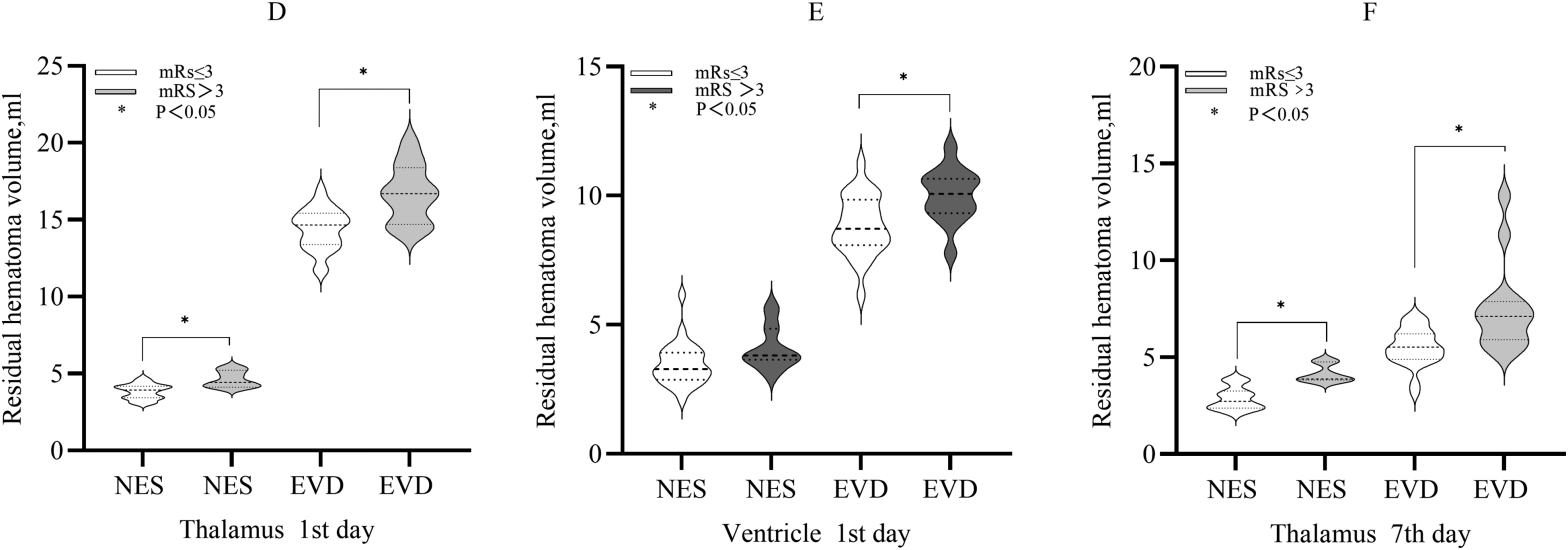
**(D)** The residual hematoma volumes in the thalamus of patients with mRS ≤3 were smaller compared with those with mRS >3 in the both NES and EVD group on the 1st day after surgery (*P* < 0.05). **(E)** The residual hematoma volume in the ventricle of patients with mRS ≤3 was smaller compared with those with mRS >3 in the EVD group on the 1st day after surgery (*P* < 0.05) **(F)**. The residual hematoma volumes in the thalamus of patients with mRS ≤3 were smaller compared with those with mRS >3 in the both NES and EVD group on the 7th day after surgery (*P* < 0.05).

Compared with patients with mRS >3, the mean residual hematoma volume in the thalamus of patients with mRS ≤3 on the 1st day was less in each group (NES: 3.82 ml vs. 4.63 ml, *P* < 0.05. EVD: 14.39 ml vs. 16.69 ml, *P* < 0.05). The median residual hematoma volume in the ventricle of patients with mRS ≤3 was less than that of patients with mRS >3 in the EVD group on the 1st day (9.01 ml vs. 10.19 ml, *P* < 0.05), and the difference was not significant in the NES group (*P* > 0.05). On the 7th day, the median residual hematoma volume in the thalamus of patients with mRS ≤3 was less compared with patients with mRS >3 in each group (NES: 2.73 ml vs. 3.87 ml, *P* < 0.05. EVD: 5.53 ml vs. 7.11 ml, *P* < 0.05) (Figure 3).

### Duration of surgery and ventilator use, blood loss, and GCS score in the two groups

**Figure 4:**
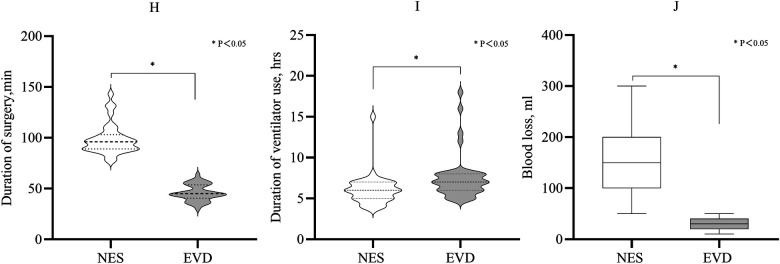
**(H)** Duration of surgery of the EVD was shorter than that of the NES group (*P* < 0.05). **(I)** The duration of ventilator use after the first surgery. It was shorter in the NES group than in the EVD group (*P* < 0.05). **(J)** The median blood loss during surgery was 150 ml and 30 ml in the NES and EVD groups, respectively. The blood loss was less in the EVD group (*P* < 0.05).

The mean duration of surgery was shorter in the EVD group than in the NES group (45 min vs. 96 min, *P* < 0.05). The duration of ventilator use in the NES group after the first surgery was shorter than that in the EVD group (median: 6 h vs. 7 h, *P* = 0.001). The median blood loss during surgery in the EVD group was less than in the NES group (30 ml vs. 150 ml, *P* < 0.05). On 3rd day after surgery, the GCS score was greater in the NES group than in the EVD group (median: 11 vs. 10, *P* = 0.033) (Figure 4; Table 2).

### Complications after surgery in the two groups

The incidence of lung infection was lower in the NES group than in the EVD group (31.43% vs. 55%, *P* < 0.05). The incidences of urinary tract infection, intracranial infection, extremity vein thrombosis, rebleeding, cerebral infraction, epilepsy, re-operation and hydrocephalus were not significantly different in the two groups (Table 2).

### Length of stay in the ICU and hospitalization duration

**Figure 5:**
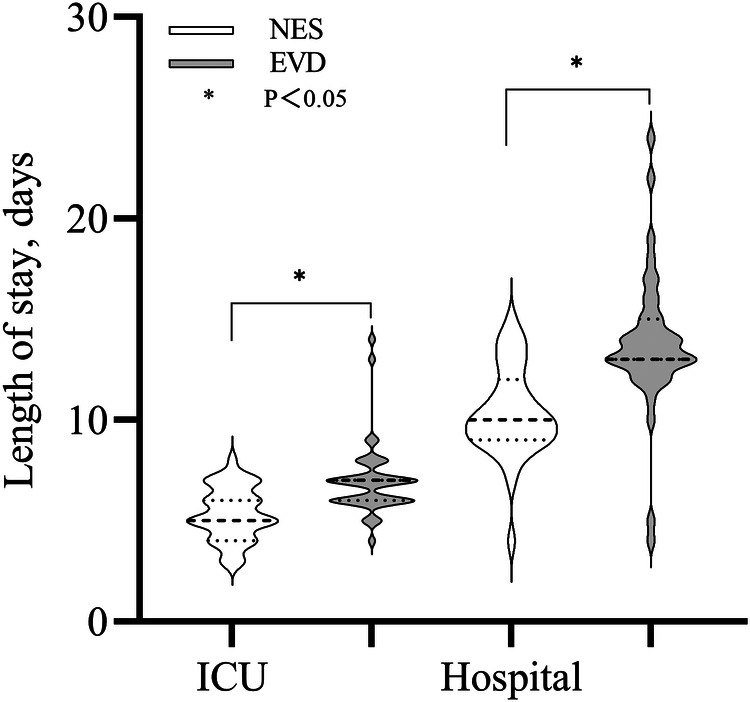
Length of stay (LOS) in the ICU and hospital were shorter in the NES group than in the EVD group (*P* < 0.05).

The median length of stay in the ICU and hospitalization duration in the NES group were 5 and 11 days, respectively. The median length of stay in the ICU and hospitalization duration in the EVD group were 7 and 13 days, respectively. The length of stay in the ICU and hospitalization duration were shorter in the NES group than in the EVD group (*P* < 0.05) (Figure 5).

## Discussion

This retrospective study aimed to determine the treatment efficacy of NES in patients with THAVH. The gross hematoma evacuation and the hematoma clearance rate were greater in the NES group than in the EVD group. The residual hematoma sizes in the thalamus and the ventricles were smaller in the NES group than in the EVD group. The residual hematoma size in the thalamus of patients with mRS ≤3 was smaller than those of patients with mRS >3 in both the NES and EVD groups on the 1st and 7th day after surgery. Within 6 months of follow-up, the patients had a better rank of modified Rankin scores in the NES group than in the EVD group (*P* < 0.05). The LOS in the ICU and hospital were shorter, and the lung infection rate was lower in the NES group. These findings implied that the efficacy of NES was superior to EVD and might improve the prognosis of patients with THAVH.

The thalamus is anatomically proximal to the mesencephalon, the ventricle, the internal capsule, and the fornix. Thalamus hemorrhage usually breaks into the ventricles, and expands to the internal capsule and the midbrain. The mass effect causes compression of the brainstem and the aqueduct, resulting in acute consciousness confusion and acute hydrocephalus (4, 10). Continuous compression or destruction of the hematoma to the internal capsule may cause permanent paralysis of the extremities (10). In addition to direct brain injury, secondary brain injuries are also caused by serious inflammatory reactions. Brain tissue can be damaged by the direct cytotoxic effects produced by the degradation of hemin (15). Heme oxygenase-1 (OH-1), a key enzyme of heme catabolism, can catalyze heme and produce free iron. Free ion is further oxidized to Fe3+, which is associated with oxidative stress, blood-brain barrier damage, brain edema, and neuronal death (15). These pathological courses are initiated within the first 6 h after cerebral hemorrhage (16, 17). Moreover, inflammatory cytokines, such as TNF-α, IL-1, and IL-10, are also significantly increased at the early stage of cerebral hemorrhage (18). These inflammatory cytokines contribute to the increase in cell apoptosis in brain tissues surrounding the hematoma (19, 20). Hence, timely hematoma evacuation is reasonable in treating thalamus hemorrhage.

External ventricular drainage (EVD) is usually used to treat intraventricular hemorrhage. This procedure can rapidly decrease intracranial pressure and drain the bloody cerebral spinal fluid (21, 22). However, it is difficult to effectively clear blood clots in the ventricle via EVD alone (23). Hence, a combination of EVD with thrombolytic drugs is used to improve hematoma clearance (24). Although these measures were adopted, the hematoma clearance rate remains lower (4). In addition, the retention time of the drainage tube is too long, which increases the risk of intracranial infection (10). Neuroendoscopic surgery can be used to effectively remove the hematoma and restore the circulation of cerebral spinal fluid (4, 13, 25). Zhou et al. conducted a study to compare the efficacy of NES and EVD in the treatment of thalamic hemorrhage with ventricle encroachment (10). The rate of hematoma clearance was 85.9 ± 5.93% in the NES group, and that was 19.8 ± 3.16% in the EVD group. This result is consistent with ours; in our study, the gross rate of hematoma clearance was 75.48% in the NES group and 18.93% in the EVD group on day 1 after surgery (*P* < 0.05). To assess the effectiveness of hematoma clearance of NES for hematomas at different sites, the hematoma clearance rates in the thalamus and the ventricle were separately calculated. In the NES group, the hematoma clearance rates in the thalamus and in the ventricle on day 1 after surgery were 75.07% and 76.01%, respectively; while they were 3.92% and 35.84% in the EVD group. The NES had a greater hematoma clearance rate of the thalamus or intraventricular hematoma than did the EVD (*P* < 0.05). Theoretically, rapid hematoma evacuation contributes to the restoration of consciousness caused by hematoma compression and increased intracranial pressure. The GCS is usually used to assess patients’ consciousness. In our study, the median GCS scores were 13 and 11 on day 3 after surgery in the NES and the EVD group, respectively. The median GCS score on day 3 was greater in the NES group than in the EVD group (*P* < 0.05). This finding reflected that the restoration of patients’ consciousness was better in the NES group than in the EVD group. In the research conducted by Zhou, the mean GCS score was 6.56 ± 0.21 on day 1 after surgery in the NES group, and that was 5.03 ± 0.25 in the EVD group. The former was greater than the latter (*P* < 0.05) (10). Our results were consistent with those of other researches (10, 13). In addition, the duration of ventilator use was also shorter in the NES group than in the EVD group (median: 6 h vs. 7 h, *P* = 0.001). This finding might indirectly support that the restoration of consciousness and respiration in the NES group was superior to the EVD group.

Theoretically, the residual hematoma is less, and the prognosis might be better. A previous study has proved that a patient with a residual hematoma size of less than 15 ml has a trend of good functional outcomes (26). In our study, the gross residual hematoma sizes were less in the NES group than in the EVD group. Patients had a better rank of mRS scores in the NES group than in the EVD group (*P* < 0.05). The results of several researches were consistent with our findings (4, 13, 25, 27). Subsequently, we analyzed the difference in patients with mRS ≤3 or mRS >3 scores in terms of the residual hematoma in the thalamus or ventricle. Patients with mRS ≤3 have smaller residual hematoma volume in the thalamus than those with mRS >3 in both the NES and EVD groups on the 1st and 7th day after surgery (*P* < 0.05). However, the difference in residual hematoma volume in the ventricle was significant only in the EVD group (*P* < 0.05). These phenomena might be explained by the hematoma clearance rate and injury of the critical peripheral nerve structure surrounding the hematoma (13). Due to a relatively lower clearance rate, a small part of the hematoma was evacuated in the ventricle on day 1 in the EVD groups, which led to a continuous injury of critical nerve structure surrounding the hematoma and resulted in a poor prognosis. Another issue should be taken seriously. Although under direct vision, an excessive hematoma evacuation might lead to iatrogenic injury of healthy brain tissue or vessels surrounding the hematoma. Hence, a blood clot, hard and tightly adhered to the brain or vessels, should not be removed roughly.

Complications also affect patient's outcomes in part. Lung infection is a common complication in patients with cerebral hemorrhage, leading to poor prognosis and increased mortality (28). In the present study, lung infection rate of patients in the NES group was 31.43%, and that was 55% in the EVD group. Patients had a lower lung infection rate in the NES group than in the EVD group (*P* < 0.05). The differences in rebleeding, intracranial infection, and epilepsy in the two groups were not statistically significant. These findings were consistent with theirs (4, 10). The rate of hydrocephalus within 6 months was lower in the NES group than in the EVD group; however, the difference was not significant (5.7% vs. 12.5%, *P* > 0.05). These findings concur with Di Rienzo's result (25).

Since more hematomas were evacuated and fewer hematomas remained, the patients’ consciousness was restored relatively rapidly in the NES group. Consequently, the length of stay in the ICU and hospitalization were shorter in the NES group (*P* < 0.05). Although the EVD was superior to the NES in terms of blood loss and surgery duration, the NES was superior to the EVD in terms of the main factors associated with prognosis, such as the hematoma clearance rate. With the advances in equipment and technique, NES will have more strengths in treating thalamus hemorrhage.

The present study has several limitations. (1) This was a retrospective study; hence, the selection of patients may result in biases. (2) The sample size was relatively small, and participants were from a single center. (3) In the present study, only the solid part of the intraventricular hematoma (60–80 Hu) that was used to represent the intraventricular hematoma volume was calculated by 3D slicer software on day 1 after surgery. Therefore, the factual hematoma volume may be underestimated in the ventricle. With these limitations, the study results should be interpreted with caution. Therefore, a well-designed prospective, randomized, multiple-center trial is essential to clarify these issues.

## Conclusions

In the present study, we separately compared the hematoma clearance in the thalamus and the ventricle, patients’ prognosis, and complications between the NES group and the EVD group. The hematoma clearance rates of both the thalamus and the ventricle in the NES group were greater than those in the EVD group. The patients who received the NES treatment had a better rank of mRS score than did those who received EVD treatment; that is, the prognosis of the former was superior to the latter. In addition, the lung infection rate was lower; the LOS in the ICU, hospitalization duration and ventilator use time were shorter in the NES group. The differences in adverse events, such as rebleeding, in the NES group were similar to those in the EVD group. These findings may support that NES is a safe and effective procedure in treating patients with thalamic hemorrhage accompanied by intraventricular hematoma.

## Data Availability

The original contributions presented in the study are included in the article/Supplementary Material, further inquiries can be directed to the corresponding author.
